# High-Performance Interfacial Solar Evaporation for Zero Liquid Discharge Treatment of Coal Chemical Concentrated Brine: Principles, Challenges, and Recent Advances

**DOI:** 10.3390/nano16040274

**Published:** 2026-02-20

**Authors:** Qing Wen, Haoyang Xiong, Chunhui Zhang, Yang Yin, Haocheng Ye, Peidong Su

**Affiliations:** School of Chemical and Environmental Engineering, China University of Mining and Technology (Beijing), Beijing 100083, China; sqt2300302065@student.cumtb.edu.cn (Q.W.); sqt2400302073@student.cumtb.edu.cn (H.X.); zhangchunhui@cumtb.edu.cn (C.Z.); 2410340128@student.cumtb.edu.cn (Y.Y.); 2310340227@student.cumtb.edu.cn (H.Y.)

**Keywords:** coal chemical concentrated brine, interfacial solar steam generation, zero liquid discharge, salt resistance

## Abstract

The rapid expansion of the coal chemical industry has led to a growing demand for effective treatment of high salinity wastewater, particularly the concentrated brine streams targeted for zero liquid discharge (ZLD) management. Conventional treatment technologies face significant challenges under such extreme conditions, underscoring the urgency of developing innovative and energy-efficient alternatives. Interfacial solar steam generation (ISSG) has emerged as a promising approach for concentrated brine treatment owing to its rapid evaporation rates, low carbon footprint, and high solar thermal energy utilization. Nevertheless, the long-term stability of solar evaporators remains limited by photothermal material degradation, excessive heat loss, and salt accumulation—all of which constitute major bottlenecks preventing large-scale implementation of ISSG in ZLD systems. This review first outlines the fundamental principles, advantages, and practical constraints of interfacial solar evaporation. It then highlights recent advances in high-performance solar evaporators featuring broadband light absorption, efficient solar thermal conversion, suppressed heat dissipation, robust anti-salt fouling behavior, and sustained operational durability. These emerging designs substantially improve the feasibility of ISSG and provide promising pathways for the clean, efficient, and sustainable treatment of concentrated brine in the coal chemical industry.

## 1. Introduction

In recent years, China’s coal chemical industry has experienced vigorous development, driven by abundant coal reserves and growing energy demand [1]. However, this industry is characterized by high water consumption, high emissions, and high pollution, and is predominantly located in regions with water scarcity and poor environmental capacity for pollutants [2,3,4]. According to statistics, the annual water consumption for coal fuel production in China reaches as high as 6.177 billion cubic meters [5]. In the coal chemical industry, converting one ton of coal into other chemical products generates approximately one cubic meter of high salinity organic wastewater [6]. Although China’s economic development model is transforming, and its energy consumption and production structures are continually being upgraded and transformed, the energy mix dominated by coal will remain a fundamental characteristic of China’s energy and chemical sector [7,8,9]. To alleviate the conflict between environmental protection and energy demand, the Ministry of Ecology and Environment has imposed stringent requirements for near-zero discharge of wastewater from the coal chemical industry [10].

**Figure 1:**
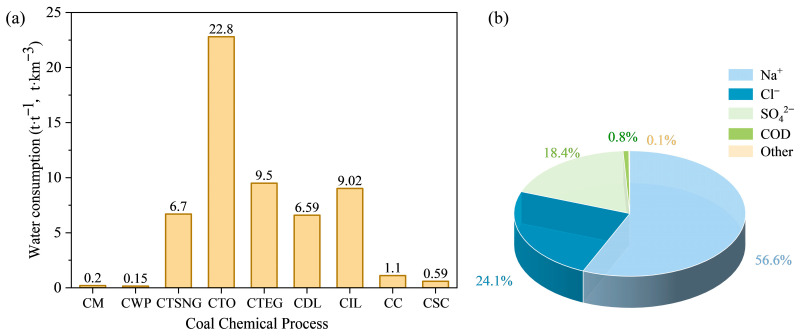
Water usage and brine composition in the coal chemical industry: (**a**) Water consumption across various coal chemical processes. Notes: CM: coal mining; CWP: coal washing and processing; CTSNG: coal-to-natural gas; CTO: coal-to-olefins; CTEG: coal-to-ethylene glycol; CDL: coal direct liquefaction; CIL: coal indirect liquefaction; CC: coal coking; CSC: coal based blue carbon [17]. (**b**) Ionic composition of concentrated brine from the coal chemical industry [18].

Coal chemical wastewater can be broadly categorized into organic wastewater and saline wastewater [11]. The salts in saline wastewater primarily originate from source water, raw coal, and chemical agents added during production processes (e.g., flocculants, acid-base neutralizers, biocides) [12]. This category mainly includes coal gasification scrubbing wastewater, reverse osmosis (RO) concentrate, ion exchange regenerant waste, discharges from demineralized water stations, circulating water blowdown, boiler blowdown, and saline wastewater generated after the biological treatment of organic wastewater [13,14]. Typically, the Total Dissolved Solids (TDS) in saline water from sources like demineralized water station discharges, circulating water blowdown, and boiler blowdown range from 3000 to 10,000 mg/L, while the TDS of RO concentrate generally reaches 10,000 to 20,000 mg/L, and often far exceeds this range [15]. As shown in Figure 1a, water consumption varies significantly across different coal chemical processes, which directly influences the magnitude of wastewater generated by each process.Wastewater with TDS reaching or exceeding the range of 1% to 3.5% (10,000–35,000 mg/L) is typically classified as high salinity wastewater. Its composition is complex, typically featuring cations such as Na^+^ and K^+^, and anions such as Cl^−^ and SO_4_^2−^. Among these, the concentrations of Na^+^, Cl^−^, and SO_4_^2−^ ions are significantly higher than those of other ions, accounting for over 90% of the total inorganic ion content [16], as illustrated in Figure 1b. Furthermore, this type of wastewater often exhibits low organic concentration, poor biodegradability, and high treatment difficulty, which constrains the achievement of zero liquid discharge in the coal chemical industry [1].

In this review, we first outline the limitations of current commercial water treatment technologies. Then we explore the emerging Interfacial Solar Steam Generation (ISSG) strategy for desalination and water purification, detailing its fundamental principles, prevailing challenges, and the material and structural design principles key to enhancing solar evaporation rates and energy conversion efficiency. Finally, we summarize its potential applications and provide a future perspective to guide subsequent research.

## 2. Current Technologies for Brine Treatment

Achieving Zero Liquid Discharge (ZLD) in treating coal chemical concentrated brine remains a formidable challenge for conventional technologies. Mainstream pressure-driven membrane processes, such as RO, are fundamentally limited for direct treatment of hypersaline streams due to exponentially increasing energy demands and a lack of membranes capable of withstanding the requisite pressures [19,20]. For instance, while seawater desalination (~35 g/L TDS) requires an applied pressure of approximately 59 bar with a thermodynamic minimum energy consumption of 2.5 kWh/m^3^, desalination of hypersaline brine (e.g., 70 g/L NaCl) demands a pressure upwards of 125 bar and a minimum energy of 6.24 kWh/m^3^. Even with advanced high pressure RO (HPRO) membranes, achieving potable water standards in a single pass remains challenging, often necessitating additional polishing steps [21]. Other desalination technologies like membrane distillation (MD) and electrodialysis (ED) cannot achieve ZLD directly [22,23,24,25,26]. Both are constrained by high capital costs, substantial energy consumption, and operational instability at high salinities, frequently leading to membrane scaling and fouling [27,28]. The extreme salinity and complex composition of such brine further undermine biological and advanced oxidation processes for organic contaminants [29,30]. While hybrid systems coupling membrane concentration with evaporative crystallization represent the conventional ZLD pathway, they incur prohibitive costs, yield low-purity mixed salts that risk secondary pollution, and face technical hurdles in efficient salt fractionation [12,31]. Consequently, developing cost-effective and sustainable ZLD strategies is imperative [32,33].

## 3. Solar-Driven Desalination Technologies

Solar energy is a promising primary energy source due to its safety, cleanliness, renewability, and widespread availability [34,35]. Leveraging this abundant and sustainable resource to drive seawater desalination presents an ideal strategy to address clean water shortages while mitigating environmental impacts [36]. Direct solar desalination technologies, which utilize solar energy to thermally produce vapour, can be functionally classified into two main categories: bulk water heating and ISSG [37].

### 3.1. Bulk Heating Solar Technology

Solar-driven evaporation offers a promising and sustainable alternative to conventional thermal processes [38]. The most traditional implementation, the solar still, employs a bulk heating method: solar energy is absorbed by a basin, which then heats the entire water volume. This leads to substantial heat loss to both the water body and the environment, resulting in low energy efficiency (typically below 40%) [39]. Consequently, this limited productivity restricts the utility of conventional solar stills for large scale applications.

### 3.2. ISSG Technology

#### 3.2.1. Basic Principle

**Figure 2:**
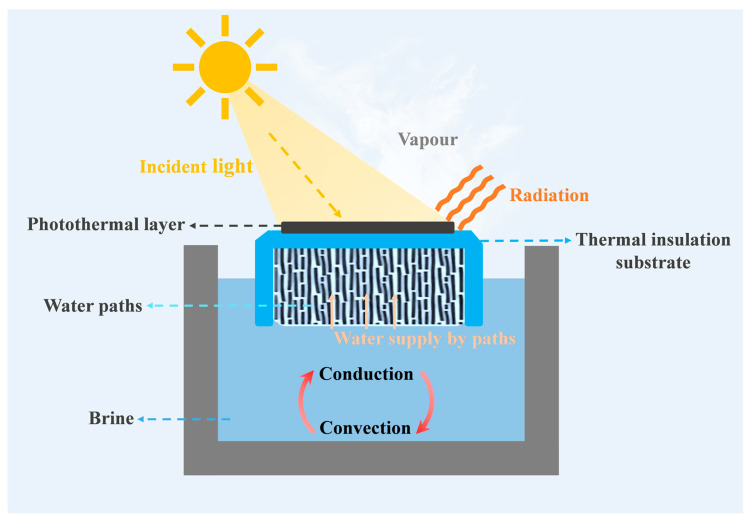
Schematic diagram of interface solar vapour generation system [43].

ISSG concentrates photothermal conversion and heat generation at the gas liquid interface, which is fundamentally different from traditional integral heating methods [40]. As shown in Figure 2, a typical ISSG structure mainly consists of a photothermal layer, water paths, and a thermal insulation substrate. The photothermal layer converts absorbed sunlight into heat [41,42]. The water paths, composed of porous structures, transport water to the photothermal layer via capillary action [43]. The thermal insulation substrate separates the photothermal conversion zone from the bulk water, ensuring that heat is used only to vaporize the water pumped to the surface, thereby avoiding unnecessary heat loss to the entire water body [44].

From a fundamental energy perspective, the performance of solar evaporators is ultimately bounded by the incident solar energy. For conventional two-dimensional (2D) interfacial evaporators under standard 1-sun illumination, the widely accepted theoretical upper limit for the evaporation rate is 1.47 kg m^−2^ h^−1^ [45]. This benchmark is derived from the ideal scenario of 100% photothermal conversion efficiency on a flat surface. However, this limit is specific to the simplified 2D model and often falls short in critically evaluating modern evaporators with complex three-dimensional (3D) architectures.

Therefore, comparing performance data requires clarifying whether the rates are based on the projected area or the actual evaporation area [46]. On this basis, the evaporation efficiency (η) should serve as the primary thermodynamic benchmark, as it intrinsically accounts for the total incident energy and enables fair comparison across different designs [47].

Recent studies report that the evaporation rates for many advanced evaporators, particularly those with 3D porous structures, significantly exceed the traditional 2D limit of 1.47 kg m^−2^ h^−1^. This enhancement primarily stems from the area gain and environmental energy gain inherent to their structural design: Firstly, the 3D porous structure effectively increases the optical path and the effective absorption area through internal light trapping effects, thereby enhancing energy capture per unit of projected area [48]. Secondly, optimized thermal localization design reduces heat loss while potentially drawing additional thermal energy from the surrounding environment [48,49]. Furthermore, the nanoscale structure and hydrophilicity of the material surface may influence the state of water molecules and the enthalpy of evaporation [50,51].

However, it must be clearly recognized that the area gain and environmental thermal gain provided by such 3D structures, along with edge effects present at small scales, can diminish significantly or even vanish when the device is scaled up—a phenomenon known as the scale effect [52]. Therefore, when evaluating such high-performance data, it is essential to specify the corresponding energy input boundaries and testing scales to avoid overestimating their actual performance in real large-scale applications.

#### 3.2.2. Key Advantages over Bulk Heating

Compared to traditional overall heating methods, ISSG has multiple advantages due to its unique heating strategy. Its core lies in concentrating heat for evaporation on the water surface, rather than heating the entire body of water, which greatly reduces heat loss and significantly improves the efficiency of converting solar energy into steam [53,54]. At the same time, this design achieves zero liquid discharge: the evaporation process occurs at the water surface interface, separating from the main saltwater, so that salt and other involatile substances do not deposit on the evaporation surface, but remain in the original solution or concentrate in specific areas for easy separation and recovery. In addition, the ISSG system structure is usually simpler and does not require complex equipment and pipelines like traditional thermal desalination. It mainly relies on solar power to drive, reducing construction and operating costs while also reducing the carbon footprint during operation [55].

#### 3.2.3. Current Challenges

Despite its promise, the transition of ISSG technology to practical, large-scale ZLD applications faces challenges. These include ensuring the long-term stability and durability of photothermal materials, managing salt accumulation to maintain evaporation performance, and further optimizing systems to achieve reliable and cost-effective ZLD for highly complex industrial brines [44,56,57].

Among these, effective salt management is a particularly critical and foundational issue. In systems reliant on passive water supply (via capillary action and convection), continuous evaporation inevitably leads to the accumulation and concentration of salt ions at the evaporation interface. This can cause supersaturation and crystallization, which can cover the photothermal surface, block water transport pathways, and thus severely compromise both system stability and evaporation performance [58].

Early solutions typically relied on physical flushing after operation cycles, which could hardly meet the practical requirements for long-term autonomous operation [59]. Therefore, more fundamental strategies have been developed. One approach aims at spatially isolating salt crystallization by designing special channels or wettability gradients to confine crystallization to predefined, non evaporative zones, thus preventing interference with the photothermal interface [60,61]. Another strategy actively prevents salt deposition on functional surfaces by tuning interfacial properties or constructing internal microstructures. These approaches aim to either transform salt crystallization into a controllable process or fundamentally suppress its adverse effects [62]. Their specific design principles and implementation methods will be systematically discussed in Section 6.1 and Section 6.2 of this article.

## 4. Application and Performance Analysis of High-Performance Photothermal Materials in ISSG

Efficient photothermal conversion is the core driving ISSG technology [63]. To maximize the capture and utilization of solar energy, developing photothermal materials with broad-spectrum strong absorption, low thermal loss, and excellent stability is crucial. Compared to traditional bulk materials, nanomaterials, due to their unique size effects (e.g., surface plasmon resonance, local field enhancement effect, tunable band gap, and high specific surface area), enable precise regulation and efficient absorption conversion of sunlight at the nanoscale, significantly enhancing photothermal performance [64,65]. These nanoscale physicochemical phenomena are key to their superiority over conventional materials [66]. Currently, widely studied nanoscale photothermal materials primarily include plasmonic metal nanoparticles, semiconductor nanomaterials, carbon nanomaterials, and emerging polymeric nanomaterials [67,68]. Given the important role of nanomaterials in advancing ISSG technology, this review will focus on representative solar absorber nanomaterials, specifically elucidating the intrinsic relationship between their nanostructure and photothermal performance.

### 4.1. Metallic Nanomaterials

Metallic nanomaterials can significantly enhance the photothermal effect through the nanoscale Localized Surface Plasmon Resonance (LSPR) mechanism [69,70]. When illuminated by sunlight at specific wavelengths, the free electrons on the surface of nanoparticles undergo collective oscillations, strongly absorbing light energy and efficiently converting it into thermal energy. This process rapidly heats the water at the interface and generates steam. Constructing micro/nanostructures for photon trapping can enhance solar absorption efficiency and promote photothermal conversion. For instance, a multiscale structured black gold membrane exhibits an absorption rate as high as 91% and a total reflectance of less than 10% across a broad spectral range of 400–2500 nm. This performance is attributed to the plasmonic nanofocusing effect induced by self-aggregated gold-coated nanowire bundle arrays [71]. Plasmonic metal nanoparticles (NPs), such as silver (Ag), gold (Au), and platinum (Pt), are widely utilized to enhance photothermal performance under low light intensity [72].

**Figure 3:**
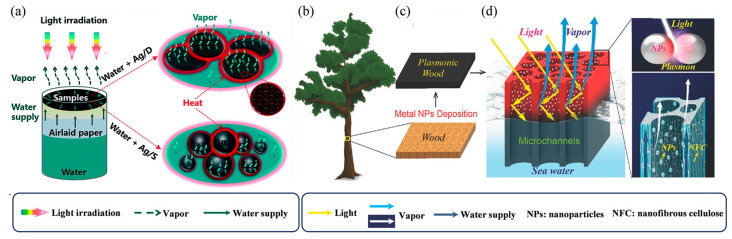
Utilization of Metallic Nanomaterials in ISSG: (**a**) Schematic of the solar vapour generation experiment using Ag/D–P–F and Ag/S–P–F. Reproduced with permission from Ref. [73]. Copyright 2017, Royal Society of Chemistry (**b**–**d**); Design of plasmonic wood: (**b**) A tree transports water from the bottom upward and absorbs sunlight for photosynthesis. (**c**) After nanoparticle decoration, the natural wood is cut perpendicular to the growth direction of the tree and turns black due to the plasmonic effect of the metal nanoparticles. (**d**) After metal nanoparticle decoration, light can be guided into the wood lumen and be fully absorbed for water vapour generation. Reproduced with permission from Ref. [74]. Copyright 2018 John Wiley and Sons.

Fang et al. developed an efficient solar steam generator based on silver nanoparticles (Ag NPs) (Figure 3a) [73]. The Ag NPs served as efficient photothermal units via the localized surface plasmon resonance (LSPR) effect. A porous diatomite substrate was employed to support and disperse the nanoparticles, maximizing their surface effects while its structure helped confine heat dissipation and promote water transport, thereby fully unlocking the photothermal potential of the Ag NPs. The evaporator demonstrated outstanding steam generation performance, achieving an evaporation efficiency of 92.2%. Zhu et al. designed a novel plasmonic material by uniformly decorating fine metal nanoparticles into the 3D mesoporous matrix of natural wood (plasmonic wood) (Figure 3b–d) [74]. Owing to the plasmonic effect of the metal nanoparticles and the waveguiding effect of the microchannels in the wood matrix, the plasmonic wood exhibited high optical absorption (≈99%) across a broad wavelength range of 200 to 2500 nm.

### 4.2. Semiconductor Materials

The photothermal performance of semiconductor nanomaterials is not a simple extension of their bulk counterparts. Nanosizing introduces the quantum confinement effect, which renders their optical properties (e.g., bandgap) tunable [75]. More importantly, precise engineering of their nanostructure—through strategies such as doping [76], alloying [75], or organic conjugate design [77]—enables the intentional creation or significant enhancement of internal defects and intermediate bands. These bands act as efficient non-radiative recombination centers, directly converting the energy of photogenerated carriers into lattice thermal energy [76]. This fundamental mechanism is the reason they achieve photothermal conversion efficiencies far surpassing those of traditional bulk materials.

Metal oxide semiconductors, extensively studied for photocatalytic reactions, are now thriving in ISSG applications. Xiong et al. reported CoCr_2_O_4_ nanocrystals rich in oxygen vacancies [78]. These vacancies were introduced via a solvothermal reduction combined with an annealing strategy, which induces a strong localized surface plasmon resonance effect (LSPR), thereby endowing the material with excellent photothermal conversion capability. The inherent hydrophilicity and the interparticle channels of the material also facilitate efficient water transport. Benefiting from this synergistic effect, the material achieved a high evaporation efficiency of 93.2%. After 30 cycles of testing in seawater, the evaporation rate exhibited a decay of less than 10%, while the crystal structure and the concentration of oxygen vacancies, the key active sites for photothermal conversion, remained stable, proving its excellent stability for practical applications. Hu’s team, for instance, developed rare earth ion (e.g., Eu^3+^)-doped SnSe nanosheets via cation exchange and liquid phase exfoliation [79]. The doping induced defect states and intermediate bands serve as efficient non-radiative recombination centers, leading to significantly enhanced light absorption and photothermal conversion. The optimized Eu^3+^-doped SnSe nanosheets (SNU) exhibit a high photothermal conversion efficiency of 82.11%. When integrated into a composite evaporator, this material enabled an evaporation rate of 2.17 kg m^−2^ h^−1^ with 96.5% efficiency under 1-sun illumination, alongside good cycling stability.

### 4.3. Carbon-Based Materials

The exceptional photothermal conversion performance of carbon nanomaterials originates from their intrinsic nanoscale sp^2^ hybridized carbon network. This structure enables broad-spectrum absorption from ultraviolet to infrared light [80,81]. Excited π electrons convert light energy into heat through efficient non-radiative relaxation, forming the physical basis for their high conversion efficiency. Beyond these inherent properties, deliberate nanostructure engineering, such as constructing hierarchical pores, rough surfaces, or 3D arrays, introduces lightNtrapping effects [82]. This pushes the macroscopic light absorption rate close to the theoretical limit. Meanwhile, their excellent nanoscale thermal conductivity, combined with rational structural design, allows for localized and efficient heat management, minimizing thermal loss [83]. Furthermore, performance can be precisely tuned at the nanoscale by means of heteroatom doping and defect engineering, enabling targeted optimization of light absorption, heat conversion, and water transport. These characteristics, governed by nanoscale structure, establish carbon nanomaterials as a superior photothermal conversion platform compared to traditional bulk carbon materials [84,85]. Representative carbon-based nanomaterials include graphene [86], graphene oxide [87], and carbon nanotubes [88].

The work by Yu et al. used nanostructure engineering to develop biomimetic hierarchical porous graphene (BHPG) for ISSG [82]. Inspired by nature, the team employed laser-induced graphene (LIG) technology to directly write porous graphene with a CO_2_ laser on a polyimide film spin coated with NaOH solution. The laser-treated sample surface exhibited microporous structures; the increased surface roughness caused multiple reflections and scattering of incident light, dually trapping the light and thereby further enhancing the light trapping capability of the graphene layer [89]. Concurrently, the NaOH activation modified the graphene surface, forming a dense porous structure and introducing hydrophilic groups, which significantly improved the material’s hydrophilicity and capillary performance. This material achieved a high evaporation rate of 2.41 kg m^−2^ h^−1^ with an efficiency of 86.6% under 1-sun illumination and demonstrated excellent salt-rejection stability. Zhao et al. further enhanced photothermal conversion efficiency by fabricating a textile evaporator via twisting carbon nanotube (CNT) fibers with cotton yarns [90]. This unique structure leveraged the nanoscale/microscale thermal conductivity difference between CNT and cotton. Their simple twisted integration created a periodic photothermal difference of approximately 5 °C at the interface. This temperature gradient induces intense Marangoni convection vapor flows around the twisted gas liquid interface, thereby enhancing water vaporization. Furthermore, the CNT material significantly disrupts the water molecule clusters within the cotton yarn, reducing the energy required for evaporation. Consequently, this textile evaporator achieved a superior evaporation rate of 2.83 kg m^−2^ h^−1^, which ranks among the highest values reported for 2D textile evaporators.

### 4.4. Polymeric Nanomaterials

The effectiveness of polymeric materials as photothermal agents is fundamentally rooted in their nanoscale design. The photothermal conversion mechanism in polymeric nanomaterials also originates from the non-radiative relaxation of delocalized π-electrons [91]. The close stacking of monomer units enhances intermolecular collisions within the polymer chains. The structure of conjugated polymers can suppress partial molecular fluorescence and augment non-radiative relaxation, thereby enabling efficient photothermal conversion [91]. Such nanostructuring endows these materials with a combination of broad-spectrum absorption, high flexibility, and tunable chemical functionality [92,93]. However, their practical application faces challenges primarily due to limited stability [94]. Currently, representative polymeric materials, including polythiophene (PT), polypyrrole (PPy), polyaniline (PAn), and polydopamine (PDA) as typical conjugated polymers, are undergoing continuous optimization of their structures and synthesis methods to overcome the stability bottleneck, thereby promoting their innovative applications in environmental protection and energy conversion.

**Figure 4:**
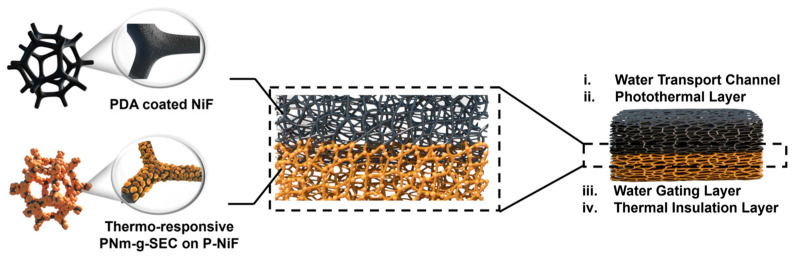
Structural diagram of bilayer evaporator SDWE. Reproduced with permission from Ref. [95]. Copyright 2024 Springer Nature.

Wang et al. designed a bilayered solar-driven water evaporator (SDWE) (Figure 4), where the upper polydopamine (PDA) layer serves as a key functional layer of polymeric nanomaterial [95]. Acting as the photothermal interface, the PDA layer combines its low thermal diffusivity with a porous nanostructure to effectively enhance light absorption, while simultaneously providing photothermal conversion functionality and nano/micro scale water transport channels. The lower layer consists of engineered sporopollenin, which functions as a thermally responsive water gate in synergy with the upper layer. This structure ensures the stable supply of a thin water film at the evaporation interface and enhances efficiency by minimizing latent heat loss. Consequently, the optimized evaporator achieved a remarkable solar utilization efficiency of 93.9% under 1-sun illumination.

### 4.5. Composite Materials

With the progression of research, investigators have begun exploring the integration of advantages from different materials to further enhance photothermal conversion efficiency and stability, leading to the emergence of composite photothermal materials [96,97,98]. Compared to single-component photothermal materials, composites leverage multi-component synergistic effects to effectively improve light absorption capacity and evaporation efficiency [99].

**Figure 5:**
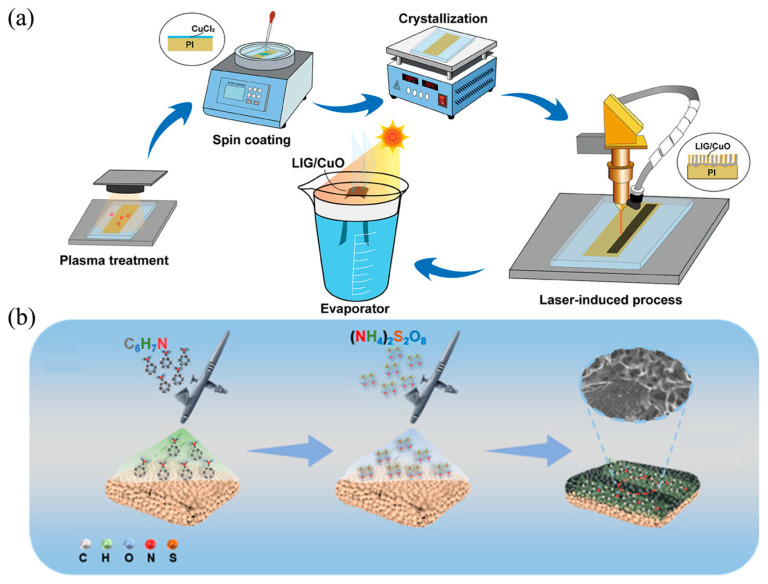
Utilization of Composite Materials in ISSG: (**a**) Fabrication process of laser-induced graphene/CuO composite on PI films coated with varying concentrations of CuCl_2_ solution using a continuous wave CO_2_ laser. Reproduced with permission from [100]. Copyright 2024, John Wiley and Sons]; (**b**) Schematic diagram of the synthesis process of LPUF−PANI. Reproduced with permission from [101]. Copyright 2024 American Chemical Society. The arrows in (**a**,**b**) indicate the sequence of the fabrication/synthesis process.

Wei et al. proposed a hydrophilic composite graphene-based material incorporating CuO (Figure 5a) [100]. The fabrication process involved coating a PI film with a CuCl_2_ solution (optimal concentration: 200 g·L^−1^), followed by exposure to continuous wave CO_2_ laser treatment. The intense local heating from the laser irradiation caused the PI film to convert into amorphous carbon. The heat generated by repeated laser scanning subsequently rearranged the carbon atoms, ultimately forming a thin layer of graphene. The CuCl_2_ served to enhance the material’s hydrophilicity by promoting the uniform distribution of CuO nanoparticles. Owing to the rapid capillary performance endowed by its hierarchical structure and enhanced hydrophilicity, the laser-induced graphene evaporator achieved an evaporation rate of 2.54 kg m^−2^ h^−1^ under 1-sun illumination with an evaporation efficiency of 91.1%, while also demonstrating excellent desalination capability. Xi et al. introduced functional lignin-based polyurethane foam (LPUF) prepared from biorefinery lignin possessing efficient photothermal properties and strong water transport capabilities (Figure 5b) [101]. Polyurethane foam (PUF) is recognized as an ideal substrate material for efficient solar water evaporation due to its low thermal conductivity, affordability, and efficient continuous water transport. However, PUF lacks inherent photothermal conversion capability and exhibits poor mechanical performance after water absorption and swelling. The incorporation of lignin effectively enhanced the mechanical properties of LPUF after swelling and endowed the foam with photothermal conversion functionality. The LPUF achieved a high moisture evaporation rate of 2.58 kg m^−2^ h^−1^. After further loading polyaniline (PANI) onto the LPUF surface, the LPUF-PANI composite maintained a stable evaporation rate above 3.0 kg m^−2^ h^−1^ across salt concentrations ranging from 3 wt% to 15 wt%, with an average evaporation rate of 3.11 kg m^−2^ h^−1^ over ten cycles. The LPUF-PANI evaporator demonstrates highly efficient evaporation and excellent stability. Furthermore, the concentrations of the four primary metal ions (Na^+^, K^+^, Mg^2+^, and Ca^2+^) in the concentrated brine were significantly lower than the World Health Organization drinking water standards both before and after the evaporation process.

**Figure 6:**
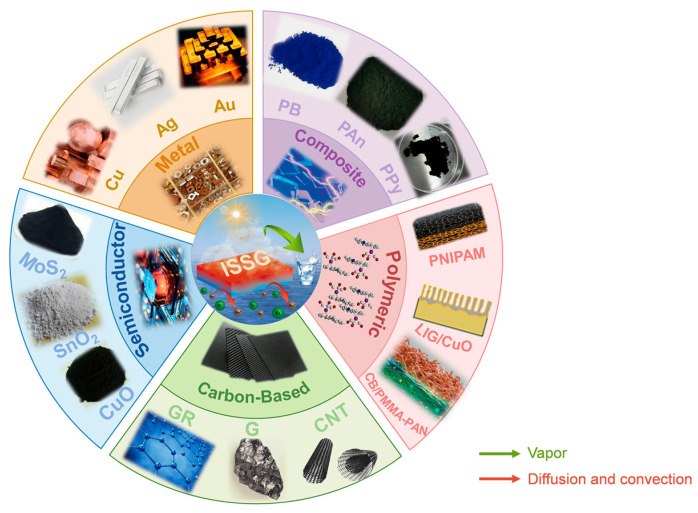
Main categories of photothermal materials in the ISSG [89,100,101,102].

The previous sections have discussed the research progress of various photothermal materials for interfacial solar evaporation. To provide a clearer overview, Figure 6 summarizes the main categories of photothermal materials used in ISSG. Based on this, Table 1 further summarizes the evaporation performance, key advantages, and current challenges of representative materials, offering readers a quick reference for understanding the material landscape in this field. As shown in Figure 6 and Table 1, different material systems have their own characteristics in terms of photothermal conversion efficiency, stability, and fabrication complexity, allowing researchers to make informed choices based on their specific needs.

## 5. Energy Regulation and Management in ISSG

### 5.1. Regulating Water Supply to Minimize Heat Loss

The matching between the system’s water supply and the solar energy input is crucial for the efficient operation of interfacial solar evaporation [107]. An imbalance between water supply and evaporation can lead to significant heat loss. In practical applications, solar irradiance fluctuates constantly, whereas the water transferred to the interface is typically constant, meaning traditional water supply systems exhibit low adaptability to changes in solar radiation density. During water supply to an interfacial solar evaporator, excessive accumulation of water at the interface can lead to bulk water heating, causing the system to revert to thin film evaporation or degrading its performance. This reduces the photothermal conversion efficiency and results in unnecessary heat loss [108].

**Figure 7:**
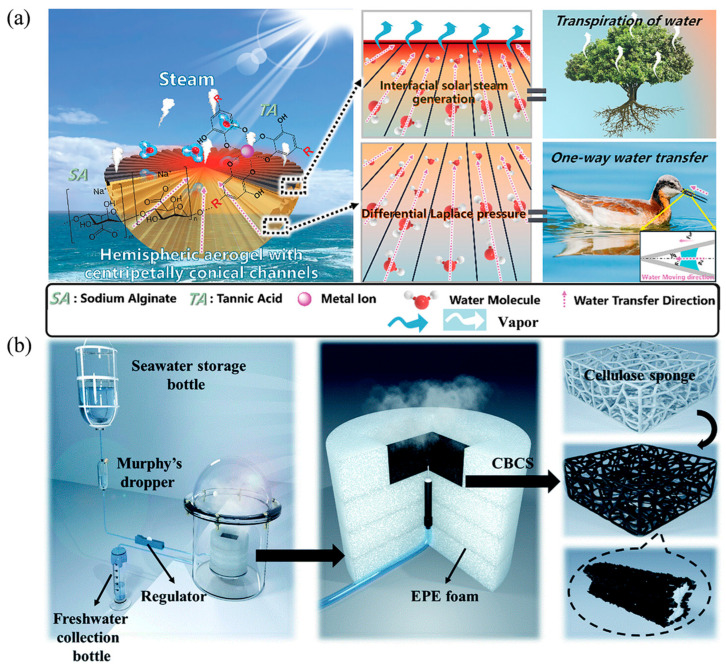
Schematic Diagrams of Interfacial Solar Evaporators with Regulated Water Supply: (**a**) Biomimetic design of STHE showcasing the detailed mechanism of water transportation in tapered channels. Reproduced with permission from [109]. Copyright 2025 John Wiley and Sons; (**b**) The CBCS structure of the enhanced carbon sponge evaporator interface evaporation system. Reproduced with permission from [110]. Copyright 2019 Royal Society of Chemistry.

Bu et al. fabricated a bionic 3D interfacial solar vapour generator (STHE) with a unidirectional water supply mechanism to further optimize water regulation and minimize heat loss (Figure 7a) [109]. Inspired by the unidirectional water transport characteristic of seabird beaks, an innovative STHE was prepared. The STHE, aligned with centripetally tapered channels, ensures unidirectional water flow and effectively suppresses downward heat transfer, thereby enhancing energy efficiency. In addition, the integration of unidirectional water supply in tapered channels with the interfacial evaporation of STHE, which mimics plant transpiration, collaboratively promotes upward water transport, achieving a reliable solar-driven water evaporation rate of about 2.26 kg m^−2^ h^−1^ under one sun irradiation. The 3D CFC Cone evaporator demonstrated excellent performance in treating wastewater (containing acid, alkali, or organic dyes) and in brine desalination. Liu et al. proposed an injection water supply system to enhance interfacial evaporation in a carbon-based sponge evaporator (CBCS) (Figure 7b) [110]. By supplying water via injection rather than absorption from a bulk water body, contact between the system and the bulk water was severed, almost entirely preventing energy dissipation into the water. Furthermore, the injected water volume could be rationally adjusted according to the incident light intensity, reducing energy loss and increasing the evaporation efficiency to approximately 91.5%, which is about 10% higher than that of traditional evaporators.

### 5.2. Reducing Enthalpy of Vaporization to Enhance Evaporation Efficiency

In recent years, the evaporation efficiency of interfacial solar evaporation has continuously improved through material upgrades and system optimization. However, limited by the enthalpy of vaporization, the evaporation efficiency of such evaporators under 1-sun illumination has long been below the thermodynamic evaporation limit: 1.47 kg m^−2^ h^−1^ [45]. Breaking free from the enthalpy limitation to achieve higher evaporation rates has become a pressing issue [111,112].

Reducing the water vaporization enthalpy through carefully designed hydrogels can unprecedentedly enhance evaporation performance under the same solar input. Hydrophilic functional groups within hydrogels can capture water molecules via hydrogen bonding to regulate water state and phase change behavior [113]. By tailoring building blocks within the hydrogel, such as functional groups/additives, polymer/monomer backbones, and crosslinkers, intermediate water can be generated within the free water and bound water gel network, leading to faster evaporation under natural sunlight [69].

Inspired by the Hofmeister effect, Wang et al. constructed a composite evaporator (CCS) consisting of a carbonized layer (photothermal layer) and a chitosan hydrogel (hydratable matrix) [114]. The core finding of this work is that Cl^−^ ions in a saline environment can specifically interact with polymer chains, disrupting their internal hydrogen bonding network, thereby significantly enhancing the hydration capacity of the hydrogel. This enhanced hydration directly increases the proportion of intermediate water in the system. Since the hydrogen bonding between intermediate water molecules is weaker than that of bulk water, the energy barrier required for evaporation is reduced. Experimental measurements confirmed that the evaporation enthalpy of the hydrogel in seawater was significantly reduced to 1397 J g^−1^, much lower than the intrinsic evaporation enthalpy of pure water (2444 J g^−1^). Thanks to this “evaporation enthalpy reduction” mechanism based on ion molecule interactions, the evaporation rate of the evaporator in saline water (3.5 wt% NaCl) under one sun’s illumination was about 25% higher than that in pure water.

Table 2 summarizes representative recent studies on energy regulation and management. The table presents key information including the material systems used in different studies, along with their evaporation efficiency and evaporation rate under 1-sun illumination.

## 6. Novel Interfacial Solar Evaporators with High Efficiency and Salt Rejection

For practical ISSG applications, particularly in achieving ZLD, managing salt deposition is a critical challenge that goes beyond merely enhancing evaporation rate and energy efficiency [69,120]. During the evaporation process, salts naturally concentrate and, without effective intervention, will form an obstructive crust on the photothermal layer. This salt crust severely impairs light absorption, blocks vapour escape, and ultimately degrades performance, hindering the transition from laboratory research to large-scale application [121,122,123]. Therefore, developing advanced salt management strategies is paramount for stable, long-term operation with concentrated brines. For high salinity streams targeted by ZLD, salt crystallization is not merely a problem to avoid but a process that can be actively managed. Recent advances have evolved two complementary strategic approaches: guiding crystallization for direct salt recovery and robustly resisting fouling to enable continuous concentration.

### 6.1. Salt Collection Strategy via Spatial Crystallization Guidance

This strategy explicitly designs systems to spatially separate salt precipitation from the evaporation zone, facilitating solid salt collection.

**Figure 8:**
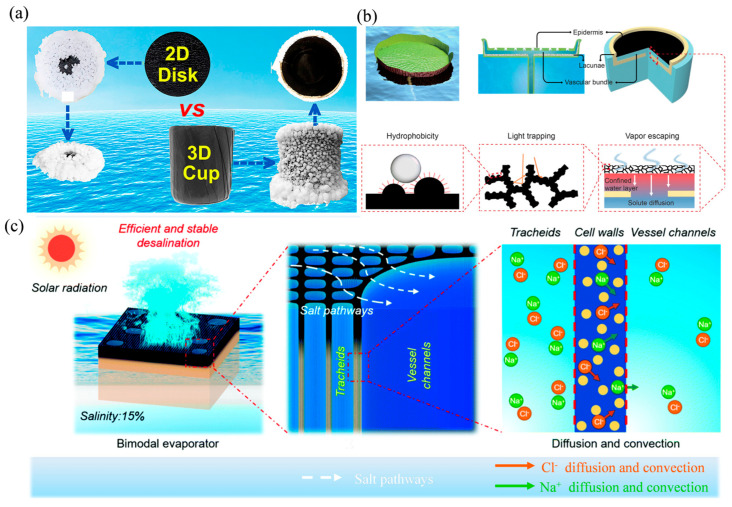
Schematic of High-Efficiency, Salt-Rejecting Interfacial Solar Evaporators. (**a**) Comparison of salt accumulation phenomena between 2D disk structure and 3D cup structure [124] Reproduced with permission from [124]. Copyright 2018 American Chemical Society; (**b**) Water lily inspired design for solar vapor generation [126] Reproduced with permission from [126]. Copyright 2019 American Association for the Advancement of Science; (**c**) Schematic of the bimodal porous balsa wood as evaporator for high salinity brine desalination. Reproduced with permission from [127]. Copyright 2019 Royal Society of Chemistry.

Shi et al. fabricated 3D cup shaped solar evaporator (Figure 8a) [124]. In this design, salt accumulation occurs exclusively on the exterior wall, thereby spatially separating the light-absorbing base from the salt precipitation surface. This prevents the evaporative surface from being blocked, allowing continuous operation with concentrated brine. Li et al. constructed 3D porous graphene spiral roll (3GSR) [125]. Its unique spiral structure induces radial capillary forces that drive brine outward, actively localizing salt crystallization to the outer regions. This design not only keeps the primary zone clear but also enables the growing salt crust to contribute to energy harvesting. The system achieved stable operation in 25 wt% brine under 1-sun illumination for 48 h, demonstrating a direct pathway toward simultaneous freshwater recovery and solid salt production.

### 6.2. Anti-Fouling Strategy via Interface and Material Design

These designs focus on preventing salt crystal deposition on the functional surface through various physicochemical mechanisms, ensuring stable long-term operation as salinity increases toward saturation.

In addition to strategies for guiding salt crystallization and recovery, a complementary design approach focuses on preventing salt deposition at the evaporation interface through innovations in materials and structure. This ensures long-term stability as brine concentration increases. For example, Wen et al. designed a zwitterionic sulfobetaine (ZSB) hydrogel evaporator, where strongly charged groups bind water molecules to form a superhydrophilic hydration layer [54]. This dynamic layer dissolves salts and carries them away, preventing surface deposition and enabling stable operation in simulated wastewater. Similarly, structural designs can actively flush salts away. Xu’s water lily inspired hierarchical structure (WHS) uses confined water channels to supply a thin layer beneath a hydrophobic absorber (Figure 8b) [126]. As vapour escapes, salts are flushed downward through the channels, maintaining a clean surface and stable evaporation efficiency even with 10 wt% brine or 30 wt% wastewater containing heavy metal ions. He et al. designed a wood evaporator with embedded artificial channel arrays (Figure 8c) [127]. By leveraging a concentration gradient, this design drives salt exchange to prevent accumulation and enable self-regeneration. A concentration gradient between natural microchannels and larger drilled channels drives rapid salt exchange through pits in the cell walls. This continuous dilution enables real-time self-regeneration, allowing stable operation for 100 h in NaCl solutions up to 20 wt%. Another effective strategy is retaining salt ions within a functional layer. Xu et al. developed a flexible Janus absorber (CB/PMMA-PAN) via electrospinning [102]. In this design, salt ions are retained in the hydrophilic PAN layer, preventing crystallization on the photothermal CB/PMMA surface. This maintains open pathways for vapour escape, providing long-term stability even in nearly saturated 20 wt% NaCl solution.

In conclusion, these advanced evaporators demonstrate that through ingenious material and structural design, salt crystallization in concentrated brines can be effectively managed—either by productively harvesting solid salt or by maintaining unobstructed evaporation. This fundamentally addresses the concern and underscores the suitability of interfacial solar evaporation for ZLD applications.

However, current research on anti-salt fouling for ISSG is predominantly focused on seawater, low salinity brine, or simplified synthetic brine (e.g., NaCl solution) [128]. While the aforementioned technologies have demonstrated promising potential in managing sodium chloride crystallization, real industrial saline wastewater (e.g., coal chemical brine) features a far more complex composition. Such streams typically contain high concentrations of scaling ions such as calcium (Ca^2+^), magnesium (Mg^2+^), and sulfate (SO_4_^2−^), along with organic contaminants [129]. During evaporation, these can form tenacious composite scales (e.g., CaSO_4_, CaCO_3_, or silica-based deposits) that are more difficult to remove, posing risks of irreversible clogging and damage to the evaporator’s photothermal surface, water transport pathways, and structural integrity [130]. Consequently, despite providing a foundational anti-salt framework, existing designs face significant challenges in directly adapting to complex real brines. Future work should prioritize developing materials and structures resistant to multi-component scaling and organic fouling, validated through long-term testing with authentic wastewater, to advance this technology toward practical industrial application.

Table 3 summarizes representative recent studies on the performance and stability of anti-salt fouling evaporators. The table presents key information including the material systems used in different studies, with a focus on their evaporation performance and long-term stability in concentrated brine.

## 7. Challenges and Progress in Long-Term Stability of ISSG Systems

### 7.1. Main Challenges to Long-Term Stability

The insufficient long-term stability of the system is a key bottleneck that restricts the practical application of ISSG technology from the laboratory, especially in the treatment of complex industrial wastewater such as coal chemical concentrated salt water. The main challenges stem from three levels: materials, interfaces, and environment. Firstly, intrinsic degradation of materials refers to the susceptibility of photothermal materials to photo corrosion, thermal aging, and chemical erosion under the synergistic effects of continous high-intensity light, local high temperatures, and complex chemical media, leading to a decline in their light absorption capacity and structural integrity. Secondly, the interface performance deteriorates. During the evaporation process, salt crystals and organic pollutants accumulate continuously at the gas liquid solid three-phase interface, forming a cover layer that seriously hinders light absorption and blocks the micro-nano channels for water supply and vapour escape. In addition, the impact of environmental fluctuations cannot be ignored. In practical application scenarios, dynamic changes in solar irradiance, fluctuations in environmental temperature and humidity, and unsteady state changes in feed brine concentration and ion composition pose continuous challenges to the system’s thermal management, water transport, and salt control capabilities, affecting its output stability and durability [130].

### 7.2. Material and Structural Design Strategies to Enhance Stability

**Figure 9:**
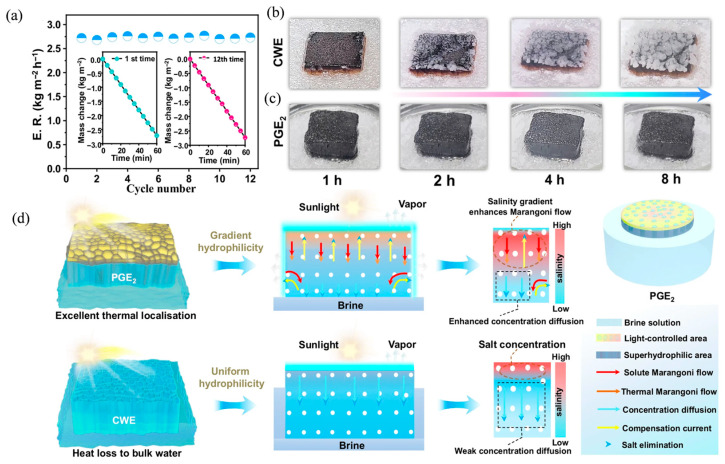
Long-term operational performance of the PGE (**a**); Evaporation rate, where the insets show the 1st and 12th mass changes, respectively (**b**,**c**); Salt-tolerance photographs and evaporation rates of CWE and PGE2 during 8 h (**d**). Comparison of Marangoni flow between PGE2 and CWE Reproduced with permission from [130]. Copyright 2025 Springer Nature.

To address the above challenges, current research is committed to achieving long-term stable operation of ISSG systems through the synergy of material design and structural innovation. At the material design level, intelligent materials that can respond to external stimuli such as light and heat can be designed to construct adaptive interfaces to cope with dynamic environments. The photocontrol dynamic water gate evaporator (PGE) developed by Wu et al. has shown significant advantages in long-term stable operation [130]. The weight of the AOA-SP coating remained unchanged, and the evaporation performance showed no significant degradation after 12 cycles (Figure 9a). The PGE maintained a stable evaporation rate throughout a 30-day outdoor experiment. In terms of anti-salt deposition and self-cleaning, the PGE achieves dynamic salt rejection through photocontrol reversible wettability transition. Even after 8 h of continuous operation in 20 wt% high-concentration brine, no significant salt crystal accumulation was observed on its surface (Figure 9b,c). This salt rejection mechanism relies not only on the hydrophilic/hydrophobic switching via SP/MC isomerization but also benefits from enhanced Marangoni flow (Figure 9d), thereby maintaining a clean evaporation interface and efficient mass transfer during long-term operation. Furthermore, the PGE exhibits strong resistance to various contaminants such as oils, acids, alkalis, and dyes, further ensuring its long-term applicability under complex water quality conditions. Deng et al. were inspired by the electromagnetic “skin effect” and designed a 3D solar evaporator with gradient porosity (SEISE) [131]. This design achieves water transport constraint through a low porosity surface layer and precisely matches the thermal constraint effect with a high porosity inner layer. This structure not only achieves a high evaporation rate, but more importantly, the rapid water transport within its surface enhances ion diffusion and convection, enabling it to operate continuously for 48 h without salt crystallization in ultra-high salinity brine (25 wt%), providing new ideas for long-term stability under extreme conditions.

## 8. Conclusions and Perspective

Interfacial solar evaporation technology has emerged as a promising, energy saving, and low-carbon strategy for managing concentrated brine, presenting a novel pathway toward achieving ZLD in the coal chemical industry. This review provides a comprehensive overview of the fundamental principles underlying this technology, surveys the latest advancements in key photothermal materials—spanning metal-based, semiconductor, carbon-based, polymeric, and composite systems—and delves into the critical roles of energy management strategies and innovative anti-salt deposition architectures in enhancing overall system performance.

Substantial progress has been made through material innovations and system engineering, exemplified by the construction of micro/nanostructures for broadband light absorption, the utilization of hydrogels to reduce water evaporation enthalpy, and the design of three-dimensional and Janus structures for directional salt rejection and self-cleaning. However, the transition of this technology from laboratory scale demonstrations to large-scale, practical implementation faces several formidable challenges that must be addressed.

A primary concern is the sustainable management of crystallized salts. While efficient water evaporation is often the central focus, the subsequent handling of residual mixed salts—including their separation, recycling, and disposal—remains largely overlooked. The development of integrated salt fractionation and crystallization processes coupled with the evaporation unit is crucial for true resource recovery and ZLD. Furthermore, the gap between idealized laboratory conditions and actual operational environments poses significant hurdles. The long-term stability, anti-fouling performance, and adaptability of these systems under fluctuating solar irradiation and complex, variable brine chemistries require rigorous validation. Another critical bottleneck lies in the efficient collection of generated vapours and the system level integration of components. Advanced condensation structures that minimize optical interference and operational resistance are essential to prevent efficiency losses and ensure high water recovery. The field would also benefit from a unified and scientifically rigorous framework for evaluating energy efficiency. Reports of “over 100%” efficiency for certain 3D evaporators highlight the need for standardized metrics to ensure comparability and credibility across studies. Finally, the inherent intermittency of sunlight necessitates the exploration of hybrid systems incorporating energy storage or complementary auxiliary power sources (e.g., industrial waste heat) to enable continuous, around-the-clock operation.

In conclusion, while interfacial solar evaporation holds immense promise for sustainable brine management in the coal chemical sector, its successful industrialization hinges on future research that systematically addresses material durability, system-level integration, economic viability, and environmental compatibility. Overcoming these barriers will require concerted multidisciplinary efforts to translate this compelling laboratory technology into practical engineering solutions that support the green transformation of the coal industry.

## Figures and Tables

**Table 1:** Summary of Photothermal Materials: Performance, Merits, and Challenges.

Category	Materials	Evaporation Efficiency (%)	Evaporation Rate * [kg m^−2^ h^−1^]	Advantages	Challenges	Refs
Metallic	Ag NPs [73]	92.2%	1.39	High photothermal efficiency Tunable spectrum Design flexibility	Insufficient chemical stability Limitation of narrowband absorption	[69,70,72,99]
	Metal nanoparticles [74]	99%	~1.18			
Semiconductors	SNU [79]	96.5%	2.17	Tunable properties Broadband absorption High stability	Limited absorption efficiency Complex synthesis	[91,103]
Carbon-based	LIG [82]	86.6%	2.41	Broadband absorption Abundant & low cost Excellent stability	Nanoparticle aggregation Complex preparation or high cost	[86,88,104]
	CNT fiber [90]		2.83			
Polymers	PPy nanowirearray fabric [105]	91.6%	2.32	Efficient photothermal conversion Tailorable structure Good flexibility Broadband absorption	Moderate photostability Limited thermal stability Limited conductivity	[90,92,93,106]
Composites	Graphene-based with CuO [100]	91.1%	2.54	Performance complementarity Integrated function	Complex fabrication Long-term stability concerns	[88]
	LPUF-PANI foam [101]		~3.11			

* Measured under 1-sun illumination (1 kW m^−2^).

**Table 2:** Summary of recent studies on energy regulation and management.

Materials	Evaporation Efficiency (%)	Evaporation Rate * [kg m^−2^ h^−1^]	Ref
Bionic 3D solar vapour generator (STHE)		2.26	[109]
Carbon-based sponge evaporator (CBCS)	91.5%		[110]
Hydrophilic/Hydrophobic Janus Aerogel	93.7%	2.14	[115]
TiO_2_@C/PAM/SA Bifunctional Hydrogel	92.13%	2.97	[116]
Bio-inspired Sandwich Evaporator	89.5%	2.67	[117]
Modified polyethylene foam evaporators (M-EPEs)	93.8%	1.497	[118]
Anisotropic porous cellulose hydrogel (APC)	90.69%	2.31	[119]

* Measured under 1-sun illumination (1 kW m^−2^).

**Table 3:** Summary of Recent Studies on the Performance and Stability of Anti-Salt Fouling Evaporators.

Materials	Performance and Stability in Concentrated Brine	Ref.
3D cup shaped solar evaporator	25 wt % without noticeable water evaporation rate decay in at least 120 h	[124]
3D porous graphene spiral roll (3GSR)	Stable 48 h in 25 wt% brine; zero liquid discharge with salt harvesting	[125]
Water lily inspired hierarchical structure (WHS)	Evaporation efficiency of ~80% out of highsalinity brine (10 wt %) and wastewater containing heavy metal ions (30 wt %)	[126]
PDMS/balsa evaporator	Outstanding stability and durability in a high salinity (15 wt%) brine	[127]
The engineered Ti_2_AlSnC nanofiber membrane evaporator	Continuous 30 days operation in concentrated acids (pH < 1) while maintaining a stable evaporation rate of 2.8 kg m^–2^ h^–1^	[128]

## Data Availability

The original contributions presented in this study are included in the article. Further inquiries can be directed to the corresponding author.
